# All-Cause Pneumonia Hospitalizations in Children <2 Years Old in Sweden, 1998 to 2012: Impact of Pneumococcal Conjugate Vaccine Introduction

**DOI:** 10.1371/journal.pone.0112211

**Published:** 2014-11-07

**Authors:** Anders Berglund, Mats Ekelund, Mark A. Fletcher, Lars Nyman

**Affiliations:** 1 Pfizer Inc., Sollentuna, Sweden; 2 Pfizer Inc., Paris, France; Centers for Disease Control & Prevention, United States of America

## Abstract

**Background:**

In late 2007, some Swedish County Councils started 7-valent pneumococcal conjugate vaccine (PCV7) implementation for children, and PCV7 was included in the national immunization program in 2009. By 2010, both PCV10 and PCV13 were licensed, and the selection of vaccine was subject to County Councils tenders. This study investigated the impact of the order of PCV introduction into vaccination programs on the incidence of all-cause pneumonia hospitalizations in children <2 years-old.

**Methods:**

Using population-based data from the publicly available National Inpatient Registry, the incidence of inpatient pneumonia (ICD-10 J12-J18) hospitalizations by County Councils among children <2 years old was identified between 1998 and 2012. Incidence rate ratios (IRR; 95% CI) were calculated during the nationwide implementation of PCV7 and then between County Councils, as based on the higher-valent vaccine chosen for a program.

**Results:**

There was a lower risk of all-cause pneumonia hospitalization among <2 year-old children following the introduction of PCV7, as compared to the pre-PCV7 period (0.77; 0.63–0.93). A decreased risk of all-cause pneumonia was also observed in the County Councils that followed the order PCV7 then PCV13 (0.82; 0.66–1.01), while no trend was observed in County Councils with a program in the order PCV7 then PCV10 (1.03; 0.82–1.30). When comparing the higher-valent vaccines, there was a 21% (0.79; 0.66–0.96) lower risk for childhood pneumonia hospitalization in County Councils finally using PCV13 as compared to the experience in County Councils that ultimately adopted PCV10.

**Conclusions:**

Among children <2 years-old, all-cause pneumonia hospitalizations were significantly reduced by 23% one to two years after introduction of PCV7 vaccination in Sweden. In those County Councils that next introduced PCV13, a further decline in all-cause pneumonia hospitalization was observed, in contrast to those County Councils that followed with PCV10; this 21% lower risk for childhood pneumonia hospitalization was statistically significant.

## Introduction

All-cause pneumonia remains a common cause of hospitalizations among infants throughout the world [1]–[3]. Since the introduction of the pneumococcal conjugate vaccines into the childhood national immunization programs (NIPs) of many countries, large reductions in all-cause pneumonia admissions have been observed, as based on experience with the 7-valent pneumococcal conjugate vaccine (PCV7) [4]–[14], which targets seven serotypes (4, 6B, 9V, 14, 18C, 19F and 23F) [15].

Recently, two higher-valent pneumococcal conjugate vaccines have become available for routine immunization of children: the 10-valent pneumococcal conjugate vaccine (PCV10), which includes three additional serotypes, 1, 5, and 7F [16], not covered by the PCV7, and the 13-valent pneumococcal conjugate vaccine (PCV13) that includes an additional six serotypes (1, 3, 5, 6A, 7F and 19A) [17].

In Sweden, PCV7 was included in the NIP for infants in 2009, although some County Councils already began implementation of PCV7 in late 2007. In 2010, both PCV10 and PCV13 were registered with a 3-dose schedule, and the selection of a higher-valent PCV was subject to County Council tenders that resulted in different choices between County Councils. The aim of the present study was to evaluate the impact of diverse PCV programs (i.e., pre-PV7 to PCV7, then either PCV7 to PCV10 or PCV7 to PCV13) on the rate of hospitalization due to all-cause pneumonia in children <2 years of age in Sweden.

In a review of the effect of PCV dosing schedules on the prevention of pneumonia, which included 37 studies of PCV7 and one study of PCV10, evidence for a reduction was found with each schedule [18]. Investigators in Nicaragua (3+0 schedule) recently observed lower rates of all-cause pneumonia hospitalizations among children after the introduction of PCV13 immunization through the GAVI Alliance [19]. Furthermore, two recent studies from Uruguay and France (both 2+1 schedules) reported a significant reduction among children <2 years old of consolidated pneumonia and community-acquired pneumonia when transitioning from PCV7 to PCV13 [20], [21].

## Material and Methods

### Data sources

**Figure 1:**
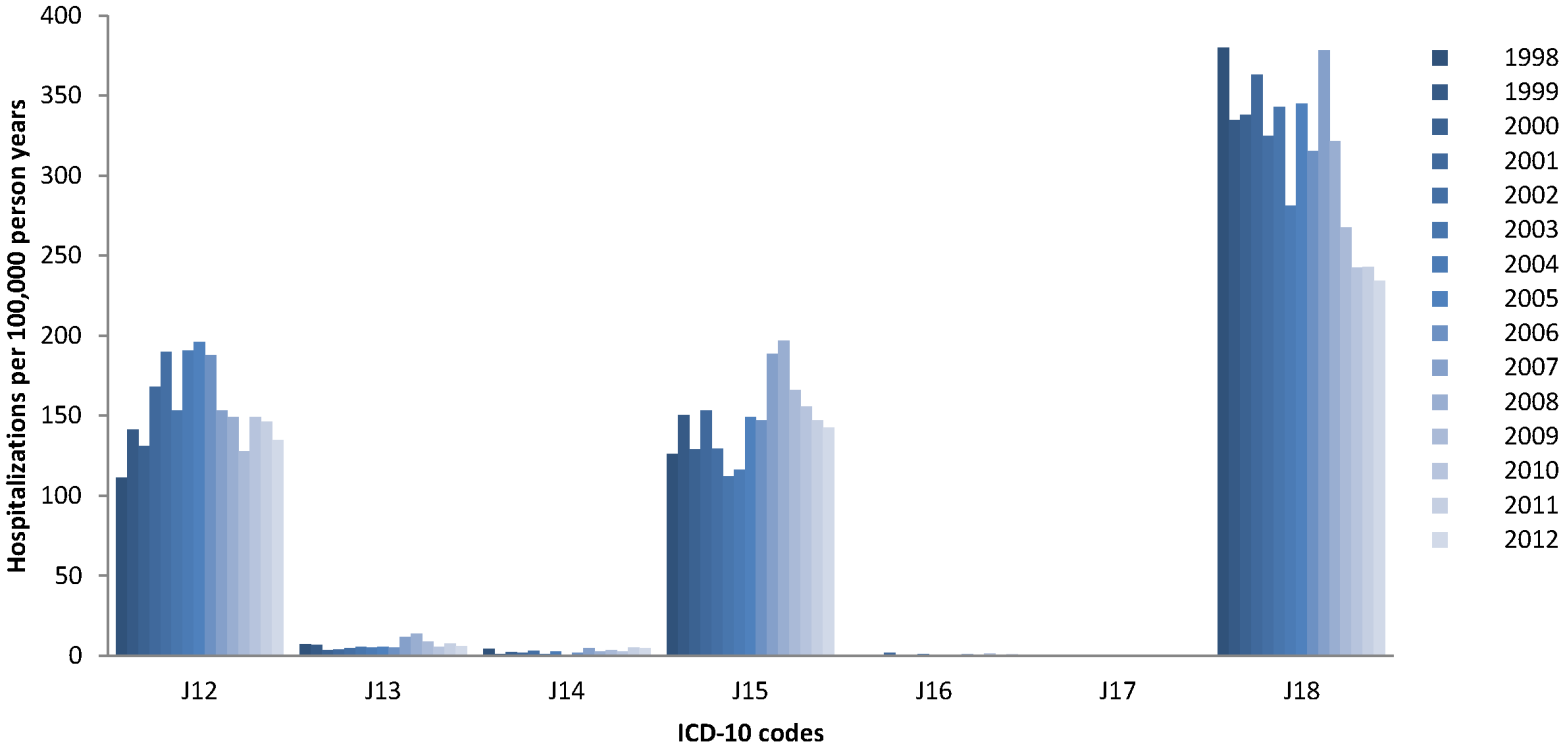
Distribution of ICD-10 code hospitalization rates by calendar year among children <2 years old: J12 (viral pneumonia), J13 (pneumonia due to Streptococcus pneumoniae), J14 (pneumonia due to Haemophilus influenzae), J15 (bacterial pneumonia), J16 (pneumonia due to other infectious organisms), J17 (pneumonia in diseases classified elsewhere), and J18 (pneumonia, unspecified organism).

The present study is a retrospective analysis of aggregated data from the publicly available National Inpatient Registry administrated by the National Board of Health and Welfare that includes nationwide information on hospital admissions and discharges since 1987 in Sweden [22], [23]. The proportion of data not properly registered in the National Inpatient Registry has been estimated to be <1% [22]. Each inpatient discharge record includes date of hospital admission, the age and sex of the patient, and discharge diagnosis coded according to the International Classification of Diseases (ICD). In the analysis, hospitalization for all-cause pneumonia was defined in accordance with the codes in the ICD-10 (J12–J18): J12 (viral pneumonia), J13 (pneumonia due to *Streptococcus pneumoniae*), J14 (pneumonia due to *Haemophilus influenzae*), J15 (bacterial pneumonia), J16 (pneumonia due to other infectious organisms), J17 (pneumonia in diseases classified elsewhere), and J18 (pneumonia, unspecified organism) **(** **Figure 1** **)**. For the present study, all-cause pneumonia admission, irrespective of the causative agent, was used, as etiology is mostly unknown in children. The current standard for making the diagnosis of pneumonia is an X-ray-confirmed infiltrate in a child with acute respiratory disease, which would include cases with a viral cause also. We have used the hospital discharge diagnosis, and although the absolute majority of these are X-ray confirmed, there is no obligate requirement to have an X-ray-confirmed infiltrate to make the diagnosis, and thus a small minority may not be confirmed.

In addition, the total number of hospital admissions for any cause of disease (i.e., all ICD-10 codes together) was identified by calendar year and County Council.

Demographic characteristics for each County Council were assembled using public information from the government agency, Statistics Sweden [24], while information on health care characteristics was retrieved from a public statistical database of the Swedish Association of Local Authorities and Regions [25].

### Study population

The number of in-patient hospital admissions for patients aged <1 year old and 1-<2 years old with one of the selected diagnosis codes (ICD-10 J12-J18) as primary diagnosis was retrieved per County Council or cluster of County Councils for each quarter in the years between 1 January 1998 and 31 December 2012. (Note that County Councils that introduced the same PCV simultaneously were clustered together.)

### The Swedish health care system and vaccine coverage

In Sweden, the 21 *Landsting* are each responsible for financing and managing the delivery of their own health care. (“County Council” is the official translation of the word for this geographical division in Sweden.)

The central government is responsible for legislation about the main principles for health care delivery, as well as guidelines for care and the monitoring of quality, and it provides additional funding to financially disadvantaged County Councils in order to ensure equitable health care for all residents in Sweden.

PCV7 was included in the Swedish NIP in January 2009 in a 2+1 infant schedule (vaccinated at 3, 5 and 12 months of age) with no catch-up, although the first County Council had already started vaccination with PCV7 in October 2007. PCV coverage for children in Sweden born in 2010 was 97.6% (range 96.4–98.6% between County Councils) [26].

Each County Council tenders PCVs independently or together with other County Councils, leading to different tender criteria and evaluation algorithms between County Councils. In 2010, the County Councils introduced either PCV10 or PCV13, although one cluster of County Councils implemented PCV7, changed to PCV13, and then changed again to PCV10. Four County Councils had PCV introduction dates that differed from all other County Councils; the populations of these County Councils are small, representing about 10% of the source population, and available data did not allow a separate analysis for each one of them. They were each clustered into pairs with the County Council that had the closest introduction date (**Table 1**).

**Table 1:** Month of implementation (in parenthesis) and vaccine, PCV7, PCV10, or PCV13, in the implementation of vaccination programs by County Council or clustered County Council and by Calendar Year.

Clustered County Councils	% of the source population	Calendar Year						
		1998–2006	2007	2008	2009	2010	2011	2012
Stockholm	25.4		(Oct) PCV7	PCV7	PCV7	(Jan) PCV13	PCV13	PCV13
Halland, VGR, Kronoberg, Gotland	22.2				(Jan) PCV7	(Jan) PCV13	PCV13	PCV13
Jönköping^1^	3.5			(April) PCV7	PCV7	(Jan) PCV13	PCV13	PCV13
Västernorrland^1^	2.2			(July) PCV7	PCV7	(Jan) PCV13	(Mar) PCV10	PCV10
Östergötland, Jämtland, Västerbotten, Norrbotten	10.2				(Jan) PCV7	(Jan) PCV13	(Mar) PCV10	PCV10
Uppsala, Västmanland, Dalarna, Gävleborg	11.2				(Jan) PCV7	(Mar) PCV10	PCV10	PCV10
Värmland, Södermanland^2^	5.1				(Jan/Oct) PCV7	PCV10	PCV10	PCV10
Skåne, Kalmar	15.9				(Jan) PCV7	(May) PCV10	PCV10	PCV10
Blekinge^3^	1.4			(Oct) PCV7	PCV7	(May) PCV10	PCV10	PCV10
Örebro^3^	2.9			(Mar) PCV7	(Oct) PCV10	PCV10	PCV10	PCV10

1 Jönköping and Västernorrland were clustered by the National Board of Health and Welfare in order to ensure confidentiality of records.

2 PCV7 was introduced in January 2009. In October 2009 the present region switched to PCV10.

3 Blekinge and Örebro were clustered by the National Board of Health and Welfare in order to ensure confidentiality of records.

The fraction of infants belonging to each age group (<1 year or 1-<2 years old) exposed to at least one dose of vaccine in a given quarter was estimated on the simplifying assumptions that the number of births is equally distributed over the calendar year and that vaccine coverage was 100%. Consequently, if a vaccination scheme began the first day of January in a given year, the fraction of infants <1 year old that has been exposed to at least one dose of vaccine will increase from 0% in the beginning of the first quarter to 25% by the end of the first quarter; consequently, 12.5% of infants on average will have been exposed to at least one dose of vaccine during this first quarter. For each additional quarter, the fraction of vaccinated infants <1 year old will increase until the maximum is reached (i.e., 75% of infants <1 years of age exposed to at least one dose of vaccine). As the first dose is given at the age of 3 months after birth, no more than 75% of <1 year-olds are exposed to at least one dose of vaccine. By the same logic, the fraction of vaccinated 1-year-old infants starts to increase one year after the introduction of a vaccine and will eventually reach 100%.

### Statistical Analyses

An additive time-series model was used to determine the trend, with seasonal and random components, by using moving averages. Trend is the long-term increase or decrease; the seasonal component relates to short-term, regular, wave-like patterns, while the random component describes the unpredictable residual fluctuations [27].

A pooled analysis combining time-series for several cross sections of clusters was applied, based on a generalized linear model for rates of hospitalization for all-cause pneumonia, using the negative binomial distribution (given that data were over dispersed) with a logarithmic link function and an offset equal to the log of the population divided by 100,000 [28], [29]. A sandwich variance estimator was used to account for possible multiple hospitalizations. Residual analyses showed no substantial deviations from model assumptions. Using negative binomial regression models, hospitalization rates per 100,000 person-years and incidence rate ratios (IRR) with corresponding 95% confidence interval (95% CI) for each explanatory variable were estimated.

The crude regression models included only the particular PCV (PCV7, PCV10 or PCV13) that was being administered at each time point, whereas the adjusted regression models also accounted for seasonal effects, calendar year, clustered County Councils, and a covariate of “mixed exposure of PCV13 and PCV10”. The variable *type of vaccine* was categorized as follows: pre-PCV7, PCV7, PCV10, or PCV13. The fundamental transition periods (e.g. pre-PCV7 to PCV7, PCV7 to PCV10, or PCV7 to PCV13) were excluded from the regression models. In a cluster of two paired County Councils, together representing 5.7% of the source population, there was a period (first quarter, 2011 to fourth quarter, 2012) with overlap where children in one of the County Councils were vaccinated with PCV10 and children in the other were vaccinated with PCV13. In the sensitivity analyses, the regression models were restricted to the same duration of time of use for PCV10 or PCV13.

All p-values were two-sided, and statistical significance was considered at p<0.05; R version 2.10.1 and STATA version 12 (STATA, College Station, Texas, USA) were used for all statistical analyses.

## Results

**Figure 2:**
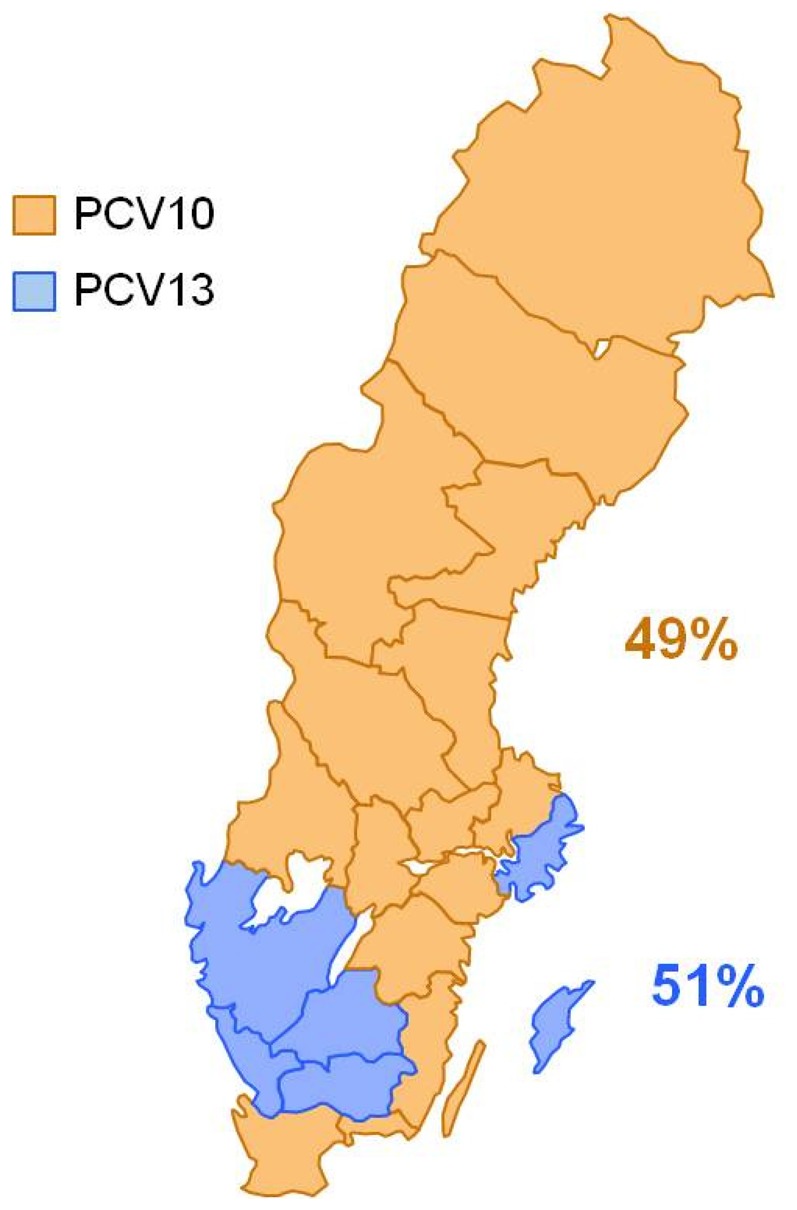
PCV use in 2012 in Sweden by County Council.

In 2012, 15 of 21 County Councils were vaccinating with PCV10 and 6 of 21 with PCV13. The County Councils vaccinating with PCV13 correspond to 51% of the source population (**Figure 2**). There were no major differences between County Councils vaccinating with PCV10 or PCV13 with regard to key demographic or health care characteristics (**Table 2**).

**Table 2:** Demographic and Health Care Characteristics Between County Councils Utilizing PCV13 and PCV10 in 2012.

	County Councils in 2012			
	Using PCV13		Using PCV10	
No. of County Councils (%)	6	(29%)	15	(71%)
No. of children <2 years (%)	115,958	(51%)	110,981	(49%)
Total population (%)	4,613,813	(48%)	4,942,080	(52%)
Share of the pop. with higher education (range)	26%	(18–30%)	21%	(17–27%)
Mean net income (>20 years) (range)^1^^,^ 2	€ 27,830	(€ 22,081–€ 29,648)	€ 23,211	(€ 21,792–€ 25,117)
Share of the pop. in urban areas (range) 3	88%	(58–96%)	82%	(66–89%)
Share of the pop. born in Sweden (range)	82%	(78–95%)	87%	(82–95%)
Mean net health care spend per inhab. (range) 1	€ 2,400	(€ 2,326–€ 2,650)	€ 2,382	(€ 2,257–€ 2,599)
Mean of hospital admissions per 1,000 (range)	166	(163–190)	170	(153–197)

1 Swedish prices converted to euros (€) by using the exchange rate of August 12, 2014 (1 Swedish kronor [SEK]  =  0.10891 euro [€]).

2 Only available in 2011.

3 Only available in 2010.

**Figure 3:**
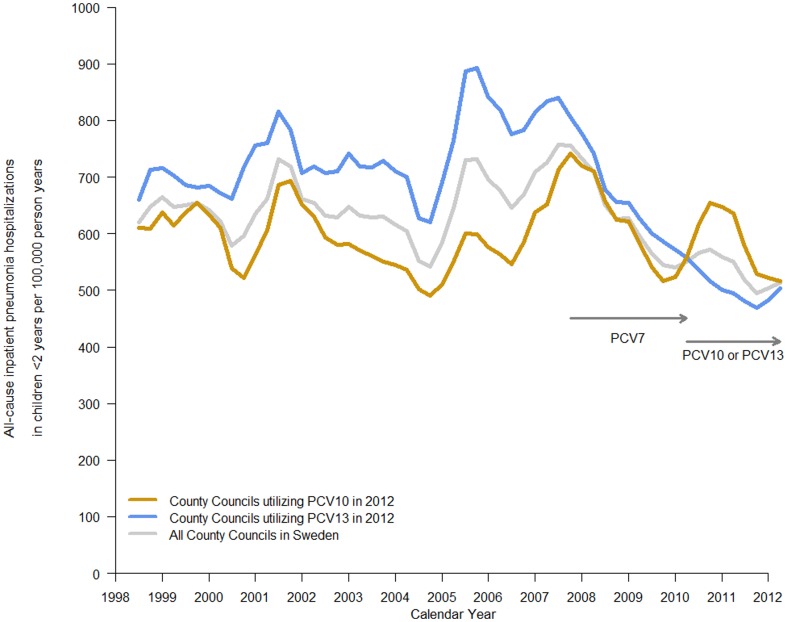
All−cause pneumonia hospitalizations per 100,000 person-years by all County Councils and by County Councils utilizing PCV10 only or PCV13 only in 2012.

A total of 37,553 hospitalizations for all-cause pneumonia among 6,013,445 person-years in children <2 years old was identified during the study period, 1998–2012. In 1998, the incidence rate per 100,000 person-years (95% CI) of all-cause pneumonia hospitalization was 615 (578–651). There was no particular trend between 1998 and 2007. A decrease commenced in 2008 and a further decrease was observed after 2009 (**Figure 3**).

In the County Councils that followed the order PCV7 then PCV13 (i.e., PCV7//PCV13), a pronounced decrease in the incidence rate was observed through 2012 (**Figure 3**); whereas for PCV7//PCV10 County Councils that introduced a program of PCV7 then PCV10, a further decrease following PCV10 was not apparent (**Figure 3**).

The crude rates of all-cause pneumonia hospitalizations per 100,000 person-years were greatest in the pre-PCV7 era (598; 568–622) and they were lowest among PCV7//PCV13 County Councils (464; 411–518) (**Table 3**). When adjustments were made for seasonal effects, calendar year, and clustered County Councils, the incidence rates for pre-PCV7, PCV7, PCV7//PCV10 County Councils and PCV7//PCV13 County Councils were 655 (619–691), 504 (414–595), 520 (440–599) and 413 (358–468), respectively.

**Table 3:** Crude and Adjusted Incidence Rates and Incidence Rate Ratios (IRR) with Corresponding 95% Confidence Intervals (CI) during the pre-PCV7 and PCV7 period, and for County Councils utilizing by 2012 PCV10 and PCV13, among children <2 years old in Sweden.

	Crude				Adjusted^1^			
Exposure variables	Rates	(95% CI)	IRR	(95% CI)	Rates	(95% CI)	IRR	(95% CI)
Pre-PCV7	597.7	(567.8–621.6)	1.00	(ref.)	654.7	(618.5–690.8)	1.00	(ref.)
PCV7	508.5	(428.8–588.2)	0.86	(0.73–1.00)	504.4	(414.1–594.6)	0.77	(0.63–0.93)
Pre-PCV7	597.7	(567.8–621.6)	1.00	(ref.)	654.7	(618.5–690.8)	1.00	(ref.)
PCV10	510.7	(444.1–577.4)	0.86	(0.75–0.98)	519.9	(440.4–599.3)	0.79	(0.68–0.93)
Pre-PCV7	597.7	(567.8–621.6)	1.00	(ref.)	654.7	(618.5–690.8)	1.00	(ref.)
PCV13	464.4	(410.9–517.9)	0.78	(0.69–0.88)	412.6	(357.5–467.6)	0.63	(0.54–0.74)
PCV7	508.5	(428.8–588.2)	1.00	(ref.)	504.4	(414.1–594.6)	1.00	(ref.)
PCV10	510.7	(444.1–577.4)	1.00	(0.82–1.23)	519.9	(440.4–599.3)	1.03	(0.82–1.30)
PCV7	508.5	(428.8–588.2)	1.00	(ref.)	504.4	(414.1–594.6)	1.00	(ref.)
PCV13	464.4	(410.9–517.9)	0.91	(0.75–1.11)	412.6	(357.5–467.6)	0.82	(0.66–1.01)
PCV10	510.7	(444.1–577.4)	1.00	(ref.)	519.9	(440.4–599.3)	1.00	(ref.)
PCV13	464.4	(410.9–517.9)	0.91	(0.77–1.08)	412.6	(357.5–467.6)	0.79	(0.66–0.96)

ref.  =  reference group.

1 The regression models were adjusted for County Councils, Seasonal effects, and Calendar Year.

In the pre-PCV7 period, the incidence rate was 584 per 100,000 person-years among County Councils that later chose PCV10 and 724 per 100,000 person-years among those that transitioned to PCV13. There was no difference in the PCV7 impact between the County Councils that eventually used PCV10 or PCV13; in particular, during the PCV7 era, the incidence decreased to 480 per 100,000 person-years in the County Councils ultimately using PCV10 and to 576 per 100,000 person-years in the PCV13 final County Councils, which was not a statistically significant difference (p = 0.233) between these groups of County Councils.

Compared with the pre-PCV7 period, there was a 23% reduced risk (0.77; 0.63–0.93) for all-cause pneumonia hospitalization in children <2 years old after PCV7 was introduced, based on a median time of follow-up of 14 months (**Table 3**). When comparing the post-PCV7 period incidence with the PCV13 period incidence, a further 18% decrease (0.82; 0.66–1.01) in the risk of hospitalizations due to all-cause pneumonia was observed in the PCV7//PCV13 County Councils, while no further decrease was observed in the PCV7//PCV10 County Councils that moved from PCV7 to a PCV10 program (1.03; 0.82–1.30).

When evaluating the rates of hospitalizations due to all-cause pneumonia among children <2 years of age in the pre-PCV7 period compared to the rates in the County Councils post-PCV10 or post-PCV13, the reduction in risk was 21% (0.79; 0.68–0.93) in the PCV7//PCV10 County Councils and 37% (0.63; 0.54–0.74) in the PCV7//PCV13 County Councils.

When contrasting the two higher-valent vaccines, a 21% (0.79; 0.66–0.96) decreased risk was observed in County Councils finally using PCV13 rather than PCV10 (**Table 3**). In analyses restricted to the same duration of time exposed to either of the higher-valent vaccines, the results remained virtually unchanged (0.78; 0.63–0.97).

## Discussion

For children <2 years of age, hospitalizations due to all-cause pneumonia were significantly reduced by 23% following the PCV7 vaccination program implemented in Sweden. In County Councils that then next chose PCV13, a trend to a further 18% decrease was observed; however, the PCV10 program that 15 County Councils followed was not associated with a further decline as compared to that already demonstrated with PCV7. In an analysis of all-cause inpatient pneumonia among <2-year-old children, there was a statistically significant 21% decreased risk among County Councils that vaccinated with PCV7 then PCV13 (PCV7//PCV13) as compared to those that followed the order PCV7//PCV10.

The introduction of PCV7 in Sweden decreased the likelihood of all-cause pneumonia hospitalizations by 23% among children <2 years old. These reductions are within the range of results from previous observational studies, where the decline in all-cause pneumonia hospitalizations in children ranged from 13–65% after implementation of PCV7 [4]–[14]. The observed reduction for Sweden is at the lower end of this spectrum, and it might be that the duration of the immunization program had been too brief to allow for the full impact of PCV7 to be observed. For instance, in the present study, for the single County Council that implemented PCV7 in 2007, ahead of the NIP, the observed reduction was 41% compared to the pre-PCV7 period (p<0.001).

A recent study in Brazil after the introduction of PCV10 as the initial pneumococcal conjugate vaccine observed a 23–29% decline of pneumonia hospitalizations (ICD-10 J12-J18) in children in 3 of 5 cities one year after implementation of PCV10, compared to an earlier period with no use of vaccines [30]. Within the first two years of a PCV13 immunization program in Nicaragua, provided as the only PCV, investigators observed statistically significant lower risks of pneumonia hospitalizations, compared to the pre-vaccination period: 33% reduction in infants <1 year-old (0.67; 0.59–0.75) and 26% in children 1-<2 years-old (0.74; 0.67–0.81) [19].

An indication of a further effect following the switch from PCV7 to PCV13 was noted on the incidence of consolidated pneumonia in Uruguay [20]. The same pattern was observed in France, where an additional 32% reduction in community-acquired pneumonia was observed after the transition from PCV7 to PCV13 among children <2 years old [21]. But to our knowledge, no other investigators have compared the rates in all-cause pneumonia based on the order of introduction of different higher-valent vaccines, PCV7 then PCV10 or PCV7 then PCV13.

The observed difference in changes of hospitalization rates attributed to the higher-valent vaccines in the present study does not seem to be related to key demographic or health care characteristics between those County Councils vaccinating with PCV10 or PCV13 (**Table 2**). The median period of time from vaccine implementation at a County Council level was 14 months for PCV7; it was 34 and 36 months for PCV10 and PCV13, respectively. In separate analyses, restricted to the same duration of time of vaccination with PCV10 or PCV13, results remained virtually unchanged with regards to a lower risk of all-cause pneumonia hospitalizations among children <2 years old in the PCV7//PCV13 County Councils. Although the start date was adjusted for in the analyses, the indirect impact of the initial PCV7 program might enlarge the impact seen in later years. Another possible reason for the observed differences in all-cause pneumonia admissions may include modifications in the criteria for pneumonia hospitalizations or the introduction of new diagnostic methods, but neither seems to have changed in recent years.

A limitation of the present study is that the data were not on an individual level. The large numbers attained using the entire Swedish population provide adequate statistical power, and the fact that the population is divided in two equally sized geographically distinct groups from different parts of the country is likely to reduce potential biases induced by regional differences, such as any variations in the annual incidence of influenza and respiratory syncytial virus hospitalizations. Nonetheless, even with two equally sized populations, we are not able to account for all residual confounding, and the observed differences between the higher-valent vaccines in the reductions of all-cause pneumonia hospitalizations may be associated with regional differences in the serotypes most frequently causing pneumococcal pneumonia or differences in the etiology of pneumonia [31].

The uptake of PCV7, PCV10 or PCV13 in the County Councils did not show any meaningful differences, and the general vaccine uptake has been very high (total 97.6%, range 96.4–98.6%). By utilizing information from the National Inpatient Registry, we were able to include all registered inpatient pneumonia hospitalizations among children aged <2 years in Sweden during the study period. Moreover, the total number of hospital admissions for all causes of disease stayed the same during the study period, suggesting a similar access to hospital beds.

A factor underlying the differences in impact between the sequence PCV7//PCV10 and PCV7//PCV13 could be the serotype coverage. Sweden has mandatory reporting for invasive pneumococcal disease (IPD) and all isolates are serotyped by the Disease Control Agency [26]. As in other countries that have introduced a pediatric vaccination program against pneumococcal disease, Sweden has noted a clear decrease in IPD among vaccinated children. For children <2 years of age, the total number of IPD cases had fallen by 75% in 2012, as compared to the average number in the years before vaccination began. In particular, the total number of IPD cases in children <2 years in all of Sweden decreased between 2010 and 2012: 26 cases in 2010, 27 cases in 2011, and 18 cases in 2012 (for which results from serotyping of 17 of 18 cases are available) [26]. The Disease Control Agency calculated the serotype coverage in children <2 years of age for the different available pneumococcal vaccines, which had remained similar between 2010 and 2012: PCV7, 12%; PCV10, 18%; PCV13, 47%. The most prevalent IPD serotypes in Sweden in 2012 for all ages were 22F (13%), 3 (13%), 7F (9%), 19A (7%), 33F (5%) and 11A (4%); data for children <2 years only are not available. The serotypes 3 and 19A are particularly interesting in this aspect because these two serotypes covered by PCV13, but not by PCV10, constitute 20% of the remaining all-age IPD burden in Sweden. It might be estimated that these serotypes also constitute an important part of the remaining IPD for children <2 years, as exemplified by the 47% coverage for PCV13, as compared to 18% for PCV10 in this same age group. This suggests that the serotypes 3 and 19A constitute a substantial part of remaining IPD burden, and should the same serotypes play a similar role in pneumonia, it may serve as an explanation for differences noted between PCV10 and PCV13 on hospitalizations due to all-cause pneumonia.

Another possible explanation may come from the transition period between different PCV programs. All children in Sweden were primed by the PCV7 with a CRM197 carrier protein conjugate against the seven targeted serotypes. Although no catch-up schedules were implemented in Sweden, County Councils used different approaches for the transitions: one County Council continued with PCV7 for all children initiated on PCV7 and in parallel started with PCV10 for new infants; others made a direct switch from PCV7 to PCV10 individually within the 2+1 schedule of the NIP [15], [16], [32]. These latter County Councils subsequently introduced PCV10 vaccine to PCV7-primed but incompletely vaccinated infants, in spite of the fact that the PCV10 has carrier proteins that are different from PCV7 for the seven serotypes in common. Other County Councils subsequently introduced PCV13 to PCV7-primed infants, where PCV13 uses the same carrier protein as PCV7 and the switch is indicated at any dose [17], [21].

## Conclusions

In conclusion, our study provides additional findings of a decreased risk of all-cause pneumonia hospitalizations among children <2 years old since implementation of the PCV7 program, as compared to the period before the pediatric NIP in Sweden. Furthermore, comparing County Councils that subsequently introduced PCV10 or PCV13, there was an additional trend of reduction of all-cause inpatient pneumonia among children <2 years old only in County Councils transitioning to PCV13. In a comparison between County Council programs that followed the order PCV7 then PCV13 or the programs that followed the order PCV7 then PCV10, there was a statistically significant 21% lower risk of all-cause inpatient pneumonia among children <2 years old in County Councils that finally vaccinated with PCV13. It is important to continue observations to understand the evolution of all-cause pneumonia hospitalizations and further document the impact of the County Council pneumococcal disease childhood immunization programs.

## References

[1] World Health Organization (2013) Immunization surveillance, assessment and monitoring. Accessed 2013 Nov 5.

[2] Wardlaw T, Salama P, Johansson EW, Mason E (2006) Pneumonia: the leading killer of children. Lancet 368(9541): 1048–50. 16997649 10.1016/S0140-6736(06)69334-3

[3] McIntosh K (2002) Community-acquired pneumonia in children. N Engl J Med 346(6): 429–37. 11832532 10.1056/NEJMra011994

[4] Grijalva CG, Nuorti JP, Arboqast PG, Martin SW, Edwards KM, et al (2007) Decline in pneumonia admissions after routine childhood immunisation with pneumococcal conjugate vaccine in the USA: a time-series analysis. Lancet 369(9568): 1179–86. 17416262 10.1016/S0140-6736(07)60564-9

[5] Griffin MR, Zhu Y, Moore MR, Whitney CG, Grijalva CG (2013) U.S. hospitalizations for pneumonia after a decade of pneumococcal vaccination. N Engl J Med 369(2): 155–63. 23841730 10.1056/NEJMoa1209165 PMC4877190

[6] Jardine A, Menzies RI, McIntyre PB (2010) Reduction in hospitalizations for pneumonia associated with the introduction of a pneumococcal conjugate vaccination schedule without a booster dose in Australia. Pediatr Infect Dis J 29(7): 607–12. 20589980 10.1097/inf.0b013e3181d7d09c

[7] Koshy E, Murray J, Bottle A, Sharland M, Saxena S (2010) Impact of the seven-valent pneumococcal conjugate vaccination (PCV7) programme on childhood hospital admissions for bacterial pneumonia and empyema in England: national time-trends study, 1997–2008. Thorax 65(9): 770–4. 20805169 10.1136/thx.2010.137802

[8] Zhou F, Kyaw MH, Shefer A, Winston CA, Nuorti JP (2007) Health care utilization for pneumonia in young children after routine pneumococcal conjugate vaccine use in the United States. Arch Pediatr Adolesc Med 161(12): 1162–8. 18056561 10.1001/archpedi.161.12.1162

[9] Simonsen L, Taylor RJ, Young-Xu Y, Haber M, May L, et al (2011) Impact of pneumococcal conjugate vaccination of infants on pneumonia and influenza hospitalization and mortality in all age groups in the United States. MBio 2(1): e00309–10. 21264063 10.1128/mBio.00309-10 PMC3025524

[10] Durando P, Crovari P, Ansaldi F, Sticchi L, Sticchi C, et al (2009) Universal childhood immunisation against Streptococcus pneumoniae: the five-year experience of Liguria Region, Italy. Vaccine 27(25–26): 3459–62. 19200823 10.1016/j.vaccine.2009.01.052

[11] Lee GE, Lorch SA, Sheffler-Collins S, Kronman MP, Shah SS (2010) National hospitalization trends for pediatric pneumonia and associated complications. Pediatrics 126(2): 204–13. 20643717 10.1542/peds.2009-3109 PMC2914815

[12] Patrzalek M, Albrecht P, Sobczynski M (2010) Significant decline in pneumonia admission rate after the introduction of routine 2+1 dose schedule heptavalent pneumococcal conjugate vaccine (PCV7) in children under 5 years of age in Kielce, Poland. Eur J Clin Microbiol Infect Dis 29(7): 787–92. 20437068 10.1007/s10096-010-0928-9

[13] Miller E, Andrews NJ, Waight PA, Slack MP, Gergoe RC (2011) Herd immunity and serotype replacement 4 years after seven-valent pneumococcal conjugate vaccination in England and Wales: an observational cohort study. Lancet Infect Dis 11(10): 760–8. 21621466 10.1016/S1473-3099(11)70090-1

[14] Rozenbaum MH, Boersma C, Postma MJ, Hak E (2011) Observed differences in invasive pneumococcal disease epidemiology after routine infant vaccination. Expert Rev Vaccines 10(2): 187–99. 21332268 10.1586/erv.10.163

[15] European Medicines Agency (2013) European Public Assessment Report, Prevenar pneumococcal saccharide conjugated vaccine (adsorbed). Accessed 2013 Dec 5.

[16] European Medicines Agency (2013) European Public Assessment Report, Synflorix pneumococcal polysaccharide conjugated vaccine (adsorbed). Accessed 2013 Dec 5.

[17] European Medicines Agency (2013) European Public Assessment Report, Prevenar 13 pneumococcal polysaccharide conjugated vaccine (13-valent, adsorbed). Accessed 2013 Dec 5.

[18] Loo JD, Conklin L, Fleming-Dutra KE, Deloria Knoll M, Park DE, et al (2014) Systematic review of the effect of pneumococcal conjugate vaccine dosing schedules on prevention of pneumonia. Pediatr Infect Dis J 33 Suppl 2: S140–51. 24336056 10.1097/INF.0000000000000082 PMC3944478

[19] Becker-Dreps S, Amaya E, Liu L, Moreno G, Rocha J, et al (2014) Changes in childhood pneumonia and infant mortality rates following introduction of the 13-valent pneumococcal conjugate vaccine in Nicaragua. Pediatr Infect Dis J 33(8): 637–42. 24445827 10.1097/INF.0000000000000269

[20] Hortal M, Estevan M, Laurani H, Iraola I, Meny M (2012) Hospitalized children with pneumonia in Uruguay: pre and post introduction of 7 and 13-valent pneumococcal conjugated vaccines into the National Immunization Program. Vaccine 30(33): 4934–8. 22664222 10.1016/j.vaccine.2012.05.054

[21] Angoulvant F, Levy C, Grimprel E, Varon E, Lorrot M, et al (2014) Early impact of 13-valent pneumococcal conjugate vaccine on community-acquired pneumonia in children. Clin Infect Dis 58(7): 918–24. 24532543 10.1093/cid/ciu006

[22] Ludvigsson JF, Andersson E, Ekbom A, Feychting M, Kim JL, et al (2011) External review and validation of the Swedish national inpatient register. BMC Public Health 11: 450. 21658213 10.1186/1471-2458-11-450 PMC3142234

[23] The National Board of Health and Welfare (2012) The statistical database on diagnosis in in-patient care. http://www.socialstyrelsen.se/statistik/statistikdatabas/diagnoserislutenvard. Accessed 3 December 2013.

[24] Statistics Sweden (2012) The statistical database for demographic characteristics in Sweden. http://scb.se/sv_/Hitta-statistik/Statistik-efter-amne. Accessed 2013 Dec 3.

[25] Swedish Association of Local Authorities and Regions (2012) The statistical database for municipalities and county councils. http://www.kolada.se. Accessed 2013 Dec 3.

[26] Swedish Institute for Communicable Disease Control (2013) Annual epidemiological report in 2012. Stockholm.

[27] Cleveland RB, Cleveland WS, McRae JE, Terpenning I (1990) STL: A seasonal-trend decomposition procedure based on loess. Journal of Official Statistics 6: 3–73.

[28] Hilbe JM (2011) Negative Binomial Regression. Cambridge: Cambridge University Press. 570 p.

[29] Frees EW (2004) Longitudinal and Panel Data. Cambridge: Cambridge University Press. 484 p.

[30] Afonso ET, Minamisava R, Bierrenbach AL, Escalante JJ, Alencar AP, et al (2013) Effect of 10-valent pneumococcal vaccine on pneumonia among children, Brazil. Emerg Infect Dis 19(4): 589–97. 23628462 10.3201/eid1904.121198 PMC3647414

[31] Fitzwater SP, Chandran A, Santosham M, Johnson HL (2012) The worldwide impact of the seven-valent pneumococcal conjugate vaccine. Pediatr Infect Dis J 31(5): 501–8. 22327872 10.1097/INF.0b013e31824de9f6

[32] World Health Organization (2012) Pneumococcal vaccines WHO position paper – 2012 – recommendations. Vaccine 30(32): 4717–4718. 22621828 10.1016/j.vaccine.2012.04.093

